# Inhibition of Oral Streptococci Growth Induced by the Complementary Action of Berberine Chloride and Antibacterial Compounds

**DOI:** 10.3390/molecules200813705

**Published:** 2015-07-28

**Authors:** Arkadiusz Dziedzic, Robert D. Wojtyczka, Robert Kubina

**Affiliations:** 1 Department of Conservative Dentistry with Endodontics, Medical University of Silesia, Pl. Akademicki 17, Bytom, 41-902 Katowice, Poland; 2 Department and Institute of Microbiology and Virology, School of Pharmacy with the Division of Laboratory Medicine in Sosnowiec, Medical University of Silesia, ul. Jagiellońska 4, Sosnowiec, 41-200 Katowice, Poland; E-Mail: rwojtyczka@sum.edu.pl; 3 Department of Pathology, School of Pharmacy with the Division of Laboratory Medicine in Sosnowiec, Medical University of Silesia, ul. Ostrogórska 30, Sosnowiec, 41-200 Katowice, Poland; E-Mail: rkubina@sum.edu.pl

**Keywords:** oral streptococci, berberine, antimicrobial activity, microdilution assay, MIC, MBC, opportunistic infection

## Abstract

Synergistic interactions between natural bioactive compounds from medicinal plants and antibiotics may exhibit therapeutic benefits, acting against oral cariogenic and opportunistic pathogens. The aim of the presented work was to assess the antibacterial activity of berberine chloride (BECl) in light of the effect exerted by common antibiotics on selected reference strains of oral streptococci (OST), and to evaluate the magnitude of interactions. Three representative oral microorganisms were investigated: *Streptococcus mutans* ATCC 25175 (SM), *S. sanguinis* ATCC 10556 (SS), *S. oralis* ATCC 9811 (SO) and microdilution tests, along with disc diffusion assays were applied. Here, we report that growth (viability) of all oral streptococci was reduced by exposure to BECl and was dependent primarily on exposure/incubation time. A minimum inhibitory concentrations (MIC) of BECl against OST ranged from 512 µg/mL (SS) to 1024 µg/mL (SM, SO). The most noticeable antibacterial effects were observed for *S. sanguinis* (MIC 512 µg/mL) and the most significant synergistic action was found for the combinations BECl-penicillin, BECl-clindamycin and BECl-erythromycin. The *S. oralis* reflects the highest MBC value as assessed by the AlamarBlue assay (2058 µg/mL). The synergy between berberine and common antibiotics demonstrates its potential use as a novel antibacterial tool for opportunistic infections and also provides a rational basis for the use of berberine as an oral hygiene measure.

## 1. Introduction

The highly diverse oral microbiota and their individual composition, including variable oral biofilms and pathogenicity, has an impact on the health and disease status of the host [1,2]. Molecular techniques have estimated the diversity within the oral cavity to consist of over 700 species or phenotypes/genotypes [3]. It is estimated that twenty-five non-homogeneous species of oral streptococci inhabit the human oral cavity and represent about 20% of the total oral bacteria [4]. A group of oral streptococci microbiota comprises cariogenic bacteria belonging to the “mutans streptococci group” and group of less or non-cariogenic bacteria co-habitating the oral cavity [5]. The relative pathogenicity of certain oral microbial species such as the mutans streptococci group (*Streptococcus mutans*, *S. mittis*, *S. sobrinus*) is undoubtfully associated with their ability to form biofilms, which are resistant to mechanical stress or antibiotic treatment [6]. Biofilm bacteria have been indicated to be up to 1000-fold more tolerant of antibiotics, and this makes it hard to treat oral streptococci with standard antimicrobials.

*S. mutans* is considered to be the primary bacterium involved in plaque formation and the initiation of dental caries and the most cariogenic of all of the oral *Streptococci* responsible for dental caries [7,8]. *Streptococcus sanguinis*, a strain of the “Viridans Streptococcus group” mostly found in dental plaque and cavities within the healthy oral mouth [9], may come to inhabit the heart valves through the bloodstream following invasive (surgical) dental procedures, leading to severe subacute bacterial endocarditis [10,11]. Treatment involves the prolonged administration of wide-spectrum antibiotics. Interestingly, *S. mutans* strain can also be a source of infective endocarditis [12]. *S. sanguinis* and mutans streptococci have an inverse relationship [13] as oral streptococci and bacteria commonly present in dental plaque may influence the viability and/or virulence of *S. mutans* [14].

*Streptococcus oralis*, another microorganism inhabiting the oral cavity, is classified as the minor opportunistic pathogen strain from the *Streptococcus mitis* group [15]. Currently, *S. oralis* is considered a potential pathogen that may affect immunocompromised patients [16] as a causative factor of several acquired health conditions such as bacterial endocarditis [17,18], respiratory diseases [19] and streptococcal septicemia [20]. *S. oralis* is known to be one of the first bacteria to colonize the pellicle on enamel and to form the plaque biofilm. Moreover, it is able to interact with common periopathogens, e.g., *Porphyromonas gingivalis*, which is closely associated with chronic periodontal disease [21]. It has emerged that *S. oralis* exhibits antimicrobial resistance towards penicillin, and its antibiotic susceptibility results are very close to the results of the other “mitis group” organisms. Montejo [22] reported *Streptococcus oralis* as a causative factor of meningitidis, a serious complication occurring following dental extraction.

The vast majority of oral streptococci strains are commensal species, but they can become pathogenic in response to host health deficiencies, systemic immune changes or local triggers, including oral hygiene deterioration and surgical interventions affecting oral health. According to recommendations of the American Heart Association [23] and the British NICE clinical guideline [24], in selected cases a systemic antibiotic prophylaxis should be administered perioperatively, prior to invasive dental procedures in order to prevent e.g., infective endocarditis, which can be linked to oral microbiota (*S. oralis*, *S. sanguinis*, and other oral streptococci) reaching the blood circulation. The antimicrobial resistance of some oral streptococci to standard synthetic penicillin and its derivatives has rendered them less effective in the treatment of opportunistic infections.

In high risk individuals, the level of oral bacteria may be reduced by pre-operative use of local measures (e.g., mouth rinses) containing antiseptic constituents, including these from natural origin. Berberine, a plant alkaloid having a long history of medicinal use in Chinese medicine, is present in a numerous medicinal plants (roots and rhizomes) such as: *Hydrastis canadensis* (goldenseal), *Coptis chinensis* (coptis or goldenthread), *Berberis aguifolium* (Oregon grape), *Berberis vulgaris* (barberry), and *Berberis aristata* [25]. Berberine extracts and their derivatives have demonstrated significant antimicrobial activity against a variety of microorganisms, including bacteria, viruses [26,27], fungi, protozoans and chlamydia [28,29]. It has been observed that berberine has weak activity against Gram-negative bacteria and is more active against Gram-positive bacteria, including S. aureus [25]. The toxicity and mutagenicity of berberine to human cells seem to be non-significant as determined in both *in vitro* and *in vivo* experiments [30].

To our knowledge, isoquinoline-type alkaloids have been under investigation in the context of their potential synergistic effects with antibiotics commonly used against oral streptococci strains. Currently, limited data exist regarding the efficacy of berberine and its derivatives that may contribute to oral health benefits [31,32]. A single report evaluated the effect of berberine derivatives on a diverse group of oral streptococci [33]. Due to the increased bacterial resistance to conventional treatment [34,35] attention is now turning to the management of infectious diseases, including opportunistic infections, with nonconventional antimicrobials.

Being aware of a common prevalence of caries, considered as a chronic worldwide disease, and potential pathogenicity of oral microbiota towards medically compromised individuals, we aimed to evaluate *in vitro* the anti-streptococcal effect of the naturally originated alkaloid berberine alone at the various concentrations and in combination with antibiotics. We generated concentration-response profiles over an experimental period of time of 24 h. The results were applied for a quantitative assessment of oral microflora growth using the most common reference oral streptococci strains (ATCC) exposed to berberine and selected antibacterial agents, including antibiotics. The antibacterial effect of berberine was evaluated by the simultaneous use of microdilution assays and a novel AlamarBlue assay.

## 2. Results and Discussion

### 2.1. Anti-Streptococcal Effect of Berberine Based on MIC/MBC Results

Our study was designed to determine the antibacterial effect of BECl and to evaluate for the first time, whether the addition of other antibiotics may augment the biological effect of this natural substance. Two subsequent susceptibility assays were used which allowed concomitant evaluation of the minimum concentration that inhibits growth of the tested oral streptococci. The MIC values obtained for each species may not represent the concentration that inhibits biofilm formation, which are more resilient than planktonic forms.

The antibacterial activity of BECl against *S. mutans* and *S. oralis* strains was uniform and comparable, with the MIC equivalent amount at 1024 µg/mL (Table 1). The lowest MIC value of 516 µg/mL was detected for *S. sanguinis*. On the contrary, *Streptococcus oralis* was two times less susceptible to the bactericidal action of BECl than *S. mutans* and *S. sanguinis*, with obtained MBC values of 2054 µg/mL and 1024 µg/mL, respectively (Table 1). Interestingly, the results of AlamarBlue assay (MIC_AB_) were not coherent with standard MIC readings (microdilution assay) and suggested a more diverse range from 1 (*S. sanguinis*) to 32 µg/mL (*S. mutans*).

**Table 1:** Mean minimum inhibitory concentrations and minimum bactericidal concentrations for BECl (reference molecule, µg/mL) assessed for a panel of oral streptococci strains.

Reference Oral Streptococcus Strain	MIC	MIC_AB_	MBC
*Streptococcus mutans* ATCC 25175	1024 µg/mL	32 µg/mL	1024 µg/mL
*Streptococcus sanguinis* ATCC 10556	512 µg/mL	1 µg/mL	1024 µg/mL
*Streptococcus oralis* ATCC 9811	1024 µg/mL	8 µg/mL	2058 µg/mL

The compound berberine chloride significantly inhibited the growth of the examined oral cariogenic and opportunistic pathogens, essentially above the concentration used of 128 µg/mL. Comparing the examined strains, *S. oralis* seems to be the most resilient bacteria species, according to the MIC/MBC microdilution assay results, while the MIC/MBC values gained from the AlamarBlue assay indicate *S. sanguinis* as a most susceptible one and *S. mutans* as the most viable cariogenic strain (MIC_AB_= 32 µg/mL). Comparison of MIC results for *S. sanguinis* and *S. oralis*, revealed *S. sanguinis* to be two times more susceptible to BECl, with obtained MIC values of 512 µg/mL and 1024 µg/mL, respectively (Table 1). There was no obvious quantitative and proportional relationship between the MIC results obtained using a standard microdilution method and the AlamarBlue assay technique. The minimum bactericidal concentrations (MBCs) evaluated for *S. mutans* and *S. sanguinis* were identical, suggesting that BECl acted in a similar bactericidal manner towards both of these strains (Table 1).

### 2.2. Time-Kill Kinetics of Oral Streptococci Growth Exposed to Different Concentrations of Berberine

The ANOVA three way multivariate analysis indicates that the growth kinetics of all oral streptococci strains were affected more by incubation time and less by berberine concentration (*p* < 0.001, Table 2). The multi-directional interactions between “strain”, “time” and “concentration” factors were statistically significant (*p* < 0.001, Table 2 and Table 3). The time of exposure to BECl (57.17%) and two-way interaction between incubation time and active substance concentration (11.44%) explained most of variance (Table 2 and Table 3). ANOVA results for AlamarBlue assay were even less “berberine-concentration sensitive”, indicating primarily the type of strain (33.42%) and incubation time (22.57%) as the main influencing factors (Table 3).

**Table 2:** Multivariate analysis of variance by three-way ANOVA of oral streptococci susceptibility to BECl expressed by standard microdilution susceptibility test. The comparisons and interactions between “strain”, “time” and “concentration” factors were statistically significant (*p* < 0.001).

Variables	df	Sum of Squares	Mean Squares	*F*	% of Variance	*p*
strain (S)	2	2.678	1.339	3328.3	12.32	<0.001
time (T)	4	12.426	3.106	7722.5	57.17	<0.001
concentration (C)	11	2.334	0.212	527.5	10.74	<0.001
SxT	8	1.909	0.239	593.3	8.78	<0.001
SxC	22	0.259	0.012	29.3	1.19	<0.001
TxC	44	1.610	0.037	91.0	7.41	<0.001
SxTxC	88	0.519	0.006	14.7	2.39	<0.001

**Table 3:** Multivariate analysis of variance by three-way ANOVA of proliferation kinetics of oral streptococci strains exposed of BECl expressed as reduction of AlamarBlue. The comparisons and interactions between “strain”, “time” and “concentration” factors were statistically significant (*p* < 0.001).

Variables	df	Sum of Squares	Mean Squares	*F*	% of Variance	*p*
strain (S)	2	136148	68074	2294.61	33.42	<0.001
time (T)	4	91946	22986	774.82	22.57	<0.001
concentration (C)	11	56632	5148	173.54	13.9	<0.001
SxT	8	38938	4867	164.06	9.56	<0.001
SxC	22	5743	261	8.80	1.41	<0.001
TxC	44	46605	1059	35.70	11.44	<0.001
SxTxC	88	31359	356	12.01	7.7	<0.001

**Figure 1:**
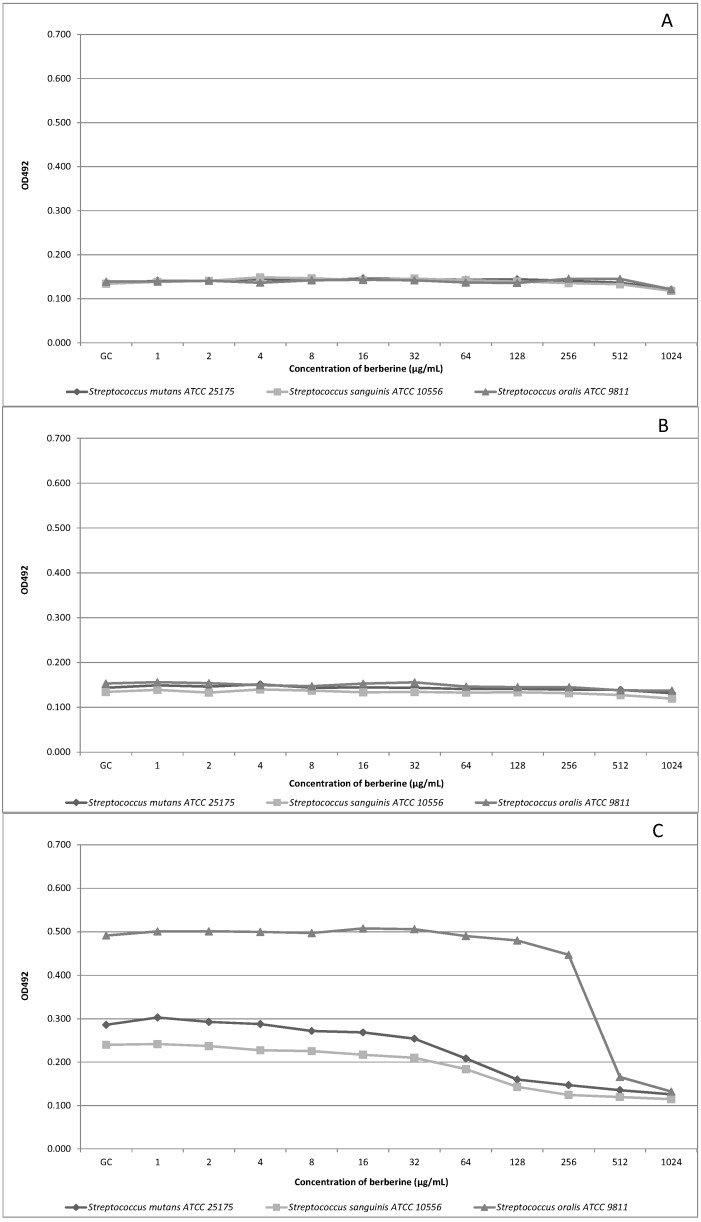

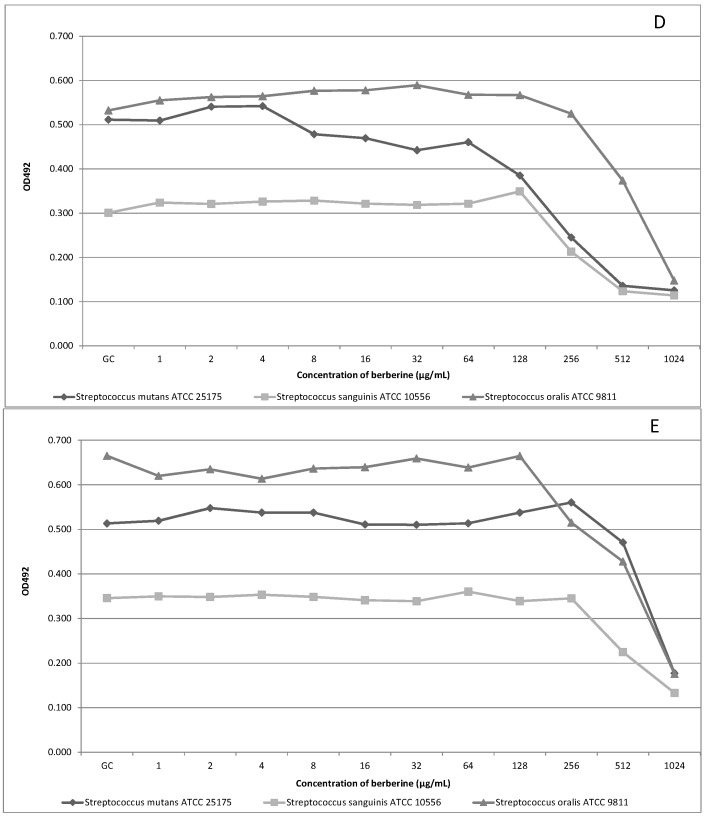
Concentration-response growth curves for oral streptococci exposed to various BECl concentrations 1–1024 µg/mL. Time-kill curves: (**A**) after 0 h of incubation; (**B**) after 2 h of incubation; (**C**) after 6 h of incubation; (**D**) after 12 h of incubation and (**E**) after 24 h of incubation.

**Figure 2:**
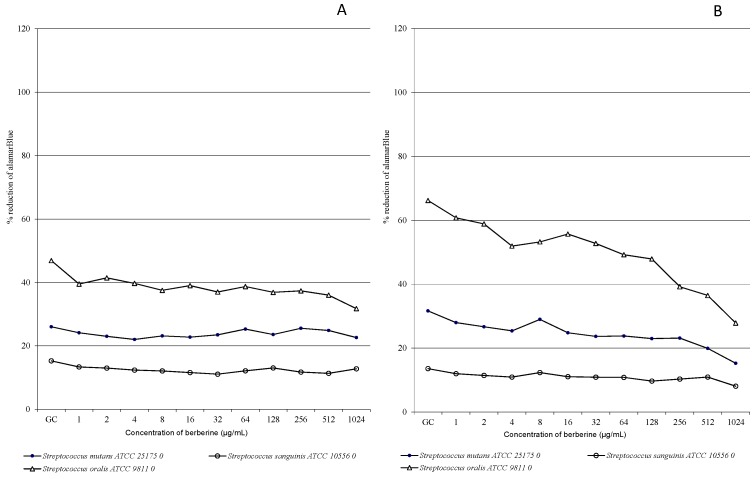

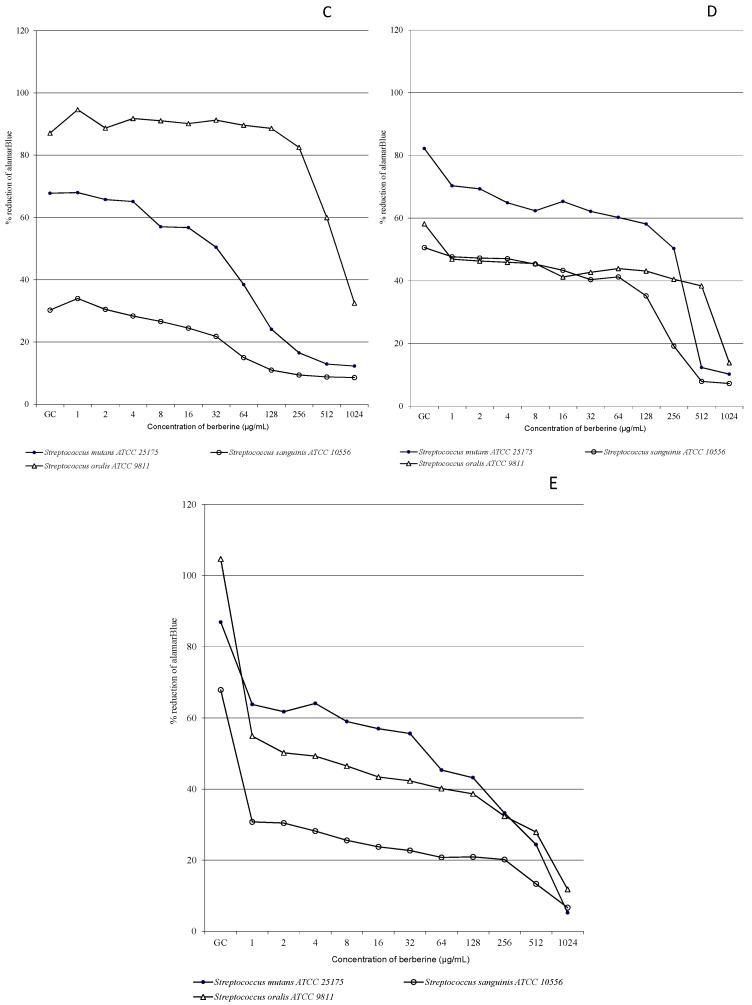
The AlamarBlue reduction ability of oral streptococci exposed to various BECl concentrations. Concentration-response growth curves: (**A**) after 0 h of incubation; (**B**) after 2 h of incubation; (**C**) after 6 h of incubation; (**D**) after 12 h of incubation and (**E**) after 24 h of incubation.

BECl concentration-response curves were used to plot the microbiological experiment results. The relative concentration-response curves obtained with BECl against a panel of oral streptococci within 24 h are shown in Figure 1A–E for the microdilution assay and in Figure 2A–E for the AlamarBlue assay. We did not observe differences between culture growth dynamics when comparing time-kill curves for experimental starting points (Figure 1A and Figure 2A). After 2 h of incubation only very slight fluctuation of the growth of the tested strains was detected, both with and without BECl addition to the medium (Figure 1B). Berberine was found to moderately suppress oral streptococci growth after 6 h of incubation and a decrease of the number of microorganisms (evidenced by OD value changes) was recorded, compared to growth control (GC, Figure 1C), above the berberine concentration of 32 µg/mL. The concentrations-curve kinetics were similar for *S. mutans* and *S. sanguinis*, contrary to the more dynamic growth of *S. oralis*. However, the *S. sanguinis* strain appears to be least susceptible to high concentrations of tested BECl (Figure 1D and Figure 2D). Concentrations-kill kinetics for the microdilution assay and AlamarBlue assay were relatively complementary for the experiment time of 6 h (Figure 1C and Figure 2C).

In the 24th h of the study (Figure 1E and Figure 2E) we observed a sudden decrease of the number of oral microorganisms compared to growth control and related to the high BECl concentration above 256 µg/mL. After 24 h of experiment (Figure 1E), up to the BECl concentrations of 128 µg/mL, a total growth inhibition was recorded for all tested strains, and no remarkable change of OD values was observed. However, the results obtained from AlamarBlue assay indicates a gradual decrease of oral streptococci growth which commenced since the starting point of the experiment and low concentrations of BECl. The reduction of oral streptococci proliferation was observed mainly for higher BECl concentrations at the end of experiment and longer incubation time (12–24 h), while lower concentrations up to 128 µg/mL seemed not to affect noticeably the growth of some strains and this phenomenon was “strain specific”. It was demonstrated that a longer treatment with berberine has a deleterious effect on oral bacteria viability as natural products are supposed to have beneficial effect after recurrent exposure frequently at low concentrations.

The data obtained for the *S. oralis* ATCC 9811 strain after 12 h of incubation (Figure 2D, AlamarBlue assay) showed that for all tested BECl concentrations up to 512 µg/mL, only a non-significant reduction of bacteria growth was observed, indicating a relative *S. oral* strain resistance to BECl. Analyzing the time-kill curves for the biofilm-forming strain *S. mutans* ATCC 25175 (Figure 1E, 24 h), a subsequent increase of the OD value was observed after 24 h for BECl concentrations ranging from 64–256 µg/mL, apparently related to the biofilm formation phenomenon. Growth and viability of oral streptococci continued to decline during exposure to BECl throughout the experimental period: 0–2–6–1–24 h. BECl was most active during the 6–12 h of exposure against *S. mutans*, and during 12–24 h against *S. mutans* and *S. sanguinis.* The bactericidal activity of BECl measured by MIC was comparable against *S. mutans* and *S. sanguinis* (Table 1). After 24 h the growth of all tested strains was not inhibited by exposure to low and moderate concentrations of berberine, although growth was significantly impaired at the highest concentration.

Our study is the second one evaluating the susceptibility of oral streptococci with the use of the novel, non-specific AlamarBlue assay [36]. This method, assessing viability kinetics, appears to be more specific considering bacteria strains and variable concentrations of evaluated active substance (Figure 1E *vs.* Figure 2E), despite of higher variation of MIC_AB_ compared to standard MIC readings (MIC_AB_ variation from 1 to 32 µg/mL). These experiments confirmed that the *S. sanguinis* and *S. oralis*. exhibit a high discrepancy between MIC and MBC results (larger MBC/MIC ratio).

### 2.3. Effects of Interaction of Berberine Chloride and Antibacterial Agents against Oral Streptococci

Next, we tested the inhibitory cumulative effect of antibiotics in the presence of BECl. A synergistic effect of suppression of oral streptococci growth was observed when berberine was used in combination with eight antibiotics: penicillin (PEN), erythromycin (ERY), clindamycin (DA), oxacillin (OXA), linezolid (LIN) and tetracycline (TET). A significant increase of the growth inhibition zone by more than 60 mm was observed around antibiotic discs after the addition of ¼ MIC BECl into the MHA medium for PEN (mean 73 mm), ERY (mean 58 mm) and DA (mean 63 mm) as shown in Table 4. These combinations resulted in a lower MIC of either active agent when measured alone. The most significant cumulative synergistic effects were detected for BECl in combination with penicillin, clindamycin and erythromycin (Table 4). For *S. oralis* strain the combination of BECl and penicillin resulted in the almost double growth inhibition zone (82 mm) including a clear reduction of the microorganisms, compared to penicillin alone (44 mm), Table 5.

**Table 4:** The combined antimicrobial effect of antibacterial agents (AB) and berberine (BECl ¼ MIC_90_) towards the reference oral streptococci strains: *S. mutans* (SM), *S. sanguinins* (SS), *S. oralis* (SO). Alteration of inhibition growth zone of tested antibiotics in combination with berberine (inhibition growth zones in mm).

AB	Inhibition Growth Zone of Antibiotic Alone (mm) SM–SS–SO	Inhibition Growth Zone of Antibiotic + BECl (mm) SM–SS–SO	Difference in Inhibition Growth Zone (mm) SM–SS–SO
PEN	43–39–48	76–61–82	33–22–34
ERY	41–43–43	56–59–61	15–26–18
DA	48–41–51	64–59–68	16–28–17
OXA	35–31–33	55–48–49	20–17–16
CIP	27–24–34	26–25–31	(−)1–1–(−)3
LIN	36–33–36	54–49–59	18–16–23
TET	37–35–41	48–45–53	11–10–12
SXT	23–21–21	26–22–24	3–1–3
mean	36.3–33.3–38.4	50.6–46–53.3	16.5–17.1–17.5

Antibacterial agent acronyms: penicillin (PEN), erythromycin (ERY), clindamycin (DA), oxacillin (OXA), ciprofloxacin (CIP), linezolid (LIN), tetracycline (TET), trimethoprim + sulfamethoxazole (SXT).

For all tested strains the combination of BECl and resulted in the appearance of a double growth inhibition zone along with a clear reduction of the microorganisms within the first inhibitory zone. Combining BECl and most of tested antibiotics produced a reduction in viability that was comparable to the viability reduction when the active agents were used alone at higher concentrations against oral microbiota strains. We did observe a slight antagonistic interaction in the case of the CIP-BECl combination for *S. mutans* and *S. oralis* (Table 5). The combination of SXT-BECl resulted in only a mild increase of antibacterial effect for all strains used in experiment (Table 5).

**Table 5:** Cumulative mean values of inhibitory growth zones for all three investigated oral streptococci strains: *S. mutans*, *S. sanguinis* and *S. oralis* (mm, SD—Standard deviation).

AB	Antibiotic Alone	Antibiotic + BECl	Mean Difference
PEN	43.33 ± 4.5 SD	73.00 ± 10.8 SD	29.67 ± 6.6 SD
ERY	42.33 ± 1.1 SD	58.67 ± 2.5 SD	19.67 ± 5.6 SD
DA	46.65 ± 5.1 SD	63.67 ± 4.4 SD	20.33 ± 6.6 SD
OXA	33.00 ± 2.0 SD	50.67 ± 3.7 SD	17.67 ± 2.0 SD
CIP	28.33 ± 5.1 SD	27.33 ± 3.2 SD	n/a
LIN	35.00 ± 1.7 SD	54.00 ± 5.0 SD	19.00 ± 3.6 SD
TET	37.67 ± 3.0 SD	48.67 ± 4.0 SD	11.00 ± 1.0 SD
SXT	21.67 ± 1.1 SD	24.00 ± 2.0 SD	2.33 ± 1.1 SD

Antibacterial agent (AB) acronyms: Penicillin (PEN), erythromycin (ERY), clindamycin (DA), oxacillin (OXA), ciprofloxacin (CIP), linezolid (LIN), tetracycline (TET), trimethoprim + sulfamethoxazole (SXT). n/a, not applicable.

The differences in growth inhibition zone were generally similar for all three investigated OST strains and the corresponding antibiotics (Table 4). The most substantial differences in growth inhibition zone size were observed for PEN (increase from 43 to 73 mm), DA (increase from 46 to 63 mm), ERY (increase from 42 to 57 mm) and LIN (increase from 35 to 54 mm). The smallest and non-significant differences in size of the growth inhibition zone were found in the presence of SXT (increase from 21 to 24 mm), TET (increase from 37 to 48 mm) and OXA (increase from 33 to 50 mm).

The most significant differences in growth inhibition zone were observed for the *Streptococcus oralis* strain (mean difference in inhibitory growth zone 17.5 mm, Table 5) and *S. oralis* was found to be the most susceptible strain towards the combined action of BECl and the investigated antibiotics (mean inhibitory growth zone 53.3 mm, Table 4). On the contrary, the weakest interactions between BECl and anti-staphylococcal drugs was found for *S. sanguinis* (mean inhibitory growth zone 46 mm). An increase of the growth inhibition zone ranging from 3 to 33 mm was observed for the *S. mutans* strain following the addition of ¼ MIC BECl into the Mueller-Hinton Agar (MHA) medium, excluding CIP.

The effect of the interaction of BECl and antibiotics on *S. sanguinis* strain was expressed as the increase of inhibition growth zone by 10–28 mm, excluding CIP and SXT. The most remarkable differences in the growth inhibition zones sizes were observed for DA, ERY and PEN, with an increase of 28 mm, 26 mm and 22 mm respectively. The values of increase of growth inhibition zones for *S. oralis* strain ranged between 12 and 34 mm, excluding CIP and SXT. The biggest discrepancy in inhibition zone growth was noted for PEN (increase from 48 to 82 mm), LIN (increase from 36 to 59 mm) and ERY (increase from 43 to 61 mm).

The antimicrobial activity of berberine is well known and has been already reported [26,27,28], along with its synergistic effect in combination with ampicillin, azithromycin, cefazolin or levofloxacin towards *S. aureus* strains [37,38] and in combination with penicillin, erythromycin, clindamycin, cefoxitin, ciprofloxacin, tobramycin, chloramphenicol, linezolid, tetracycline towards *S. epidermidis* strains [39]. Reports about the synergistic effects of different compounds on oral streptococci are rare [40], however they indicate the possibility of augmenting the antibacterial effect of commonly used antibiotics by adding certain natural compounds. Our results showed that the most noticeable synergistic effect was observed for BECl in combination with penicillin and clindamycin (Table 4), the most commonly used therapeutics recommended for prophylactic antibiotic cover in patients with high risk of infective endocarditis.

To the best our knowledge the presented work seems to be a pioneering study focused on the antibacterial effect of BECl alone and in combination with the selected antibiotics on oral streptococci, other than *S. mutans.* To date there are limited references disclosing the enhanced effect observed when berberine is used in combine action with conventional antimicrobial agents against oral opportunistic pathogens. Here, we illustrate that use of berberine salt in combination with standard antibiotics exhibits a complementary, anti-streptococcal effect towards tested OST strains. Obtained data were partially coherent with the results from other studies. A mild differences in susceptibility to BECl were observed among the test species. Nevertheless, we did not observe a clear relationship between BECl concentrations and diminishment of oral streptococci growth, however the relatively high concentration values above 256 µg/mL elicited an obvious anti-streptococcal effect.

These findings stressed the likelihood of clinical significance of the MIC/MBC ratio alone in accounting for the relative difficulty in treating of opportunistic infections caused by oral streptococci. Perhaps, other factors, such as the lower absolute sensitivity of *S. oralis* compared to that of *S. sanguinis*, must be playing a role as both the MIC and the MBC values were significantly higher for *S. oralis*. This is particularly important, taking into account the fact of existence of amoxicillin-resistant oral streptococci identified in dental plaque specimens and oral cavity [41,42] which are likely to be causative factor of infective endocarditis in susceptible individuals. The clinical findings from the studies of Masuda *et al.* [43] and Nemoto *et al.* [44] showed a higher prevalence of ampicillin-resistant strains in children at risk for infective endocarditis as compared to generally healthy population. They concluded that alternative antibiotics should be considered for such individuals when prescribing prophylaxis procedures.

According to previous reports, oral streptococci strains were found to show a relatively variable susceptibility to antibiotics and other active substances with natural origin [45,46]. The alkaloid berberine, isolated from the medicinal plant *Coptidis rhizoma* (*Ranunculacea*) from China, demonstrated antimicrobial activity against seven periodontal pathogens [31]. The authors stated that berberine evoked bactericidal activity against oral bacteria, with substantial activity against *A. actinomycetemcomitans* (MIC = 13 μg/mL) and *Porphyromonas gingivalis* (MIC = 20 μg/mL). Remarkably less activity was observed against *Lactobacillus* and *Streptococcus species*, which is complementary with our results (minimum MIC value 512 μg/mL, maximum 1024 μg/mL). Berberine also inhibited the collagenase activity of *A. actinomycetemcomitans* and *P. gingivalis* [31]. In an *in vitro* study [47], Xie *et al.* evaluated the antimicrobial efficacy of berberine solution against selected endodontic pathogens using a biofilm tooth model. The minimal inhibitory concentration of berberine against *Fusobacterium nucleatum*, *Prevotella intermedia*, and *Enterococcus faecalis* was significantly lower than the values obtained by us for oral streptococci as the MIC’s were: 31.25 μg/mL, 3.8 μg/mL, and 500 μg/mL, respectively. Berberine (2 mg/μL), when combined with 1% chlorhexidine was comparable in bactericidal activity with 5.25% sodium hypochlorite and 2% chlorhexidine.

Scazzocchio *et al.* [48] reported the antimicrobial efficiency of berberine against *S. sanguinis* and other bacteria, including its cariostatic effect. Roher *et al.* [32] investigated *in vitro* the bacteriostatic and bactericidal activities of a *Mahonia aquifolium* extract and two of major alkaloids, berberine chloride and oxyacanthine sulphate, against nine different oral bacteria. Minimum inhibitory concentrations were in the range from 0.002% to >0.125% for berberine chloride. Like our results (excluding *S. oralis*), the values for the minimum bactericidal concentrations fell in the same range, indicating that the tested substances likely acted in a bactericidal manner. The results of this study have shown that berberine and combinations were effective at inhibiting biofilm formation of all the test species, except the periopathogen *A. actinomycetemcomitans*. Contrary to our results, all concentrations of berberine revealed anti-growth effect towards bacterial strains.

Kinghorn *et al.* [40] observed a synergistic inhibition of oral pathogens growth, including *S. mutans*, when berberine was tested in combination with chlorhexidine gluconate and the antibiotics tetracycline hydrochloride or doxycycline hydrochloride. This augmented effect resulted in a lower minimum inhibitory concentration compared to the MIC of either agent when investigated alone. *Streptococcus mutans* strain exhibited lowest susceptibility to berberine, with MIC value 125 µg/mL—eight to sixteen times higher than that of the periopathogens. Reduced viability of *S. mutans* was dependent on concentration and exposure time. The inventors of a barberine-containing preparation proposed a novel clinical application for berberine in the treatment of periodontal diseases [40].

A model of the synergistic and augmented action of BECl with commonly used antimicrobial agents is believed to be attributable to interference with bacterial resistance modalities, including the supression/inhibition of the bacterial multi drug resistance (MDR) efflux pump [49]. Resistance to antibiotics hypothetically is related to the MDR efflux pumps in bacterial cell membranes and efficient removal of the antibacterial agents. Berberine, by accumulating in the bacteria cells may prevent the MDR pump from eliminating some antibiotics and thus potentiate the antibiotics’ action. [50]. Based on previous reports, the possible mechanisms of berberine’s bactericidal effect may include destruction of the bacterial cell structure, diminished DNA replication and/or RNA transcription, and binding of proteins in the biofilm, interrupting its stability [51]. Recent studies confirmed that berberine may act as a moderate inhibitor of filamenting temperature-sensitive mutant Z (FtsZ), an essential and highly conserved bacterial cytokinesis protein responsible for bacterial cell division [52].

The presented results demonstrate potentiated interactions between berberine and antibiotics as a novel tool for the management of some opportunistic infections and oral health problems with oral streptococci etiology. Moreover, the augmented use of berberine as a local measure and adjuvant antibiotic may be considered as a novel preventative modality against, e.g., infective endocarditis, with relation to invasive dental procedures, in immunocompromised individuals, particularly pediatric and adult patients with leukemia and other malignant hematological conditions. Currently, the global population is facing an increasing number of elderly and medically compromised individuals who are potentially at risk for developing severe respiratory infections due to aspiration of microbes from the oral cavity and throat. The routine use of antimicrobial mouth rinses may be effective in preventing dental plaque accumulation when used in addition to the mechanical control of plaque. Further studies should be implemented to validate how the berberine interacts with the other active anti-streptococcal agents. The authors’ next research aim shall be the determination of clinical effectiveness of other natural substances based on the results gathered from clinical studies, including the evaluation of susceptibility of a complex opportunistic oral microenvironment.

## 3. Experimental Section

### 3.1. Oral Bacteria Strains, Media and Reagents

**Figure 3:**
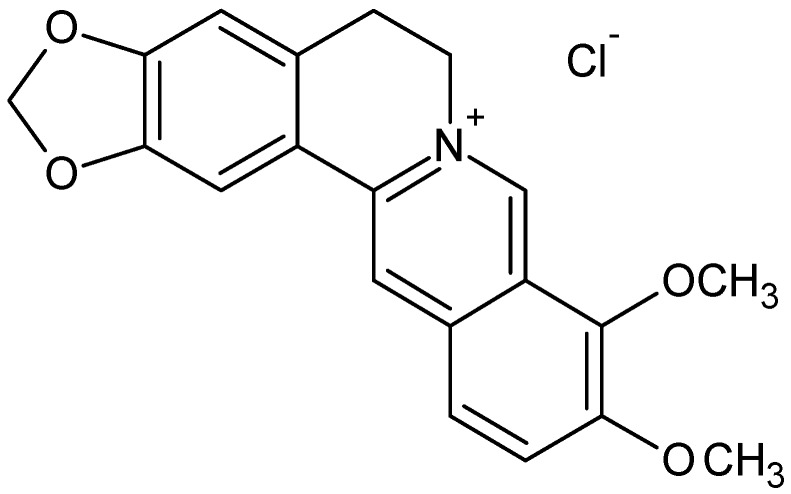
Chemical (molecular) structure of the berberine derivative berberine chloride salt, containing a protoberberine skeleton and nitrogen cation ion. Modifications of the berberine molecule leads to its derivatives exhibiting valuable pharmacological properties.

The biological activity of the natural alkaloid berberine was investigated towards the three cariogenic oral streptococci reference strains: *Streptococcus mutans* ATCC 25175 (isolated from carious dentin), *Streptococcus sanguinis* ATCC 10556 (isolated from a patient with subacute bacterial endocarditis) and *Streptococcus oralis* ATCC 9811 (isolated from a human mouth). Cultures were obtained from American Type Culture Collection (Manassas, VA, USA), biological resource center. Bacterial strains were stored for further analyses in Tryptic Soy Broth (TSB) medium with 20% of glycerol at −80 °C and used as required. TSB and Mueller-Hinton Agar (MHA) supplemented with 5% sheep blood were obtained from (BTL, Łódź, Poland). Berberine chloride (C_20_H_18_ClNO_4_, molecular weight 371.81, Figure 3) was purchased from Sigma Chemical Co. (St. Louis, MO, USA). Berberine, formulated as a chloride salt which eliminates solubility problems associated with the original plant extract compounds, was dissolved in deionized water and filtered through a 0.22 µm Millipore filter (Sartorius Co., Bohemia, NY, USA) before use.

### 3.2. Microdilution Method of Determination of Minimal Inhibiting Concentration and Minimal Bactericidal Concentration for Oral Streptococci

The minimum inhibitory concentrations (MICs) of berberine chloride (BECl) towards the oral strains were measured using the standard microdilution liquid method in sterile Nunc 96-well polystyrene plates using brain-heart infusion (BHI) medium and growth inhibition method, in a final volume of 200 μL [53,54]. The cell concentrations were estimated from the optical densities at 600 nm wavelength with the formula CFU/mL = *A*_600_ (3.8 × 10^8^), where CFU was the number of colony-forming units. One hundred microliters of mid-logarithmic-phase bacterial cultures (5 × 10^5^ CFU/mL) in Brain Heart Infusion broth (BHI) was added to 100 μL of serially diluted BECl (1024–1 µg/mL). Wells containing BHI with bacterial inoculum only served as the bacterial growth control (GC). Additional controls included BHI alone (medium sterility control), BHI with different concentrations of BECl and bacterial inoculum. All samples were prepared in triplicates. Microplates were incubated at 37 °C for 20 h, with CO_2_ supplementation (microaerophilic conditions) and the bacterial cell growth was assessed by measuring the optical density of cultures at 600 nm wavelength with a Multiskan EX microplate reader (Thermo Electron Corp., Waltham, MA, USA) [55].

The MICs were defined as the lowest concentration that completely inhibits bacterial growth [54]. The MBCs were expressed as the lowest concentration of an antimicrobial agent (mg/L), that *in vitro* reduces the number of bacteria by 99.9%, within a defined period of time [56]. To determine the MBC value of BECl 100 µL aliquots from each BECl dilution, were transferred into MHA plates and incubated at 37 °C for 20 h. After incubation period, the number of colonies was calculated, and the initial CFU/well retrospectively determined [57].

### 3.3. AlamarBlue Susceptibility/Viability Colorimetric Assay

Additionally, the AlamarBlue planktonic susceptibility/viability assay of examined strains was performed by the reference broth microdilution assay, using round-bottom, polystyrene, non-tissue culture-treated microtiter plates and BHI medium. After 24 h, 5 µL AlamarBlue was added to the wells (105 µL total volume) and the plates were shaken gently and incubated for 1 h at 37 °C. Plates were gently shaken again, and absorbances at 570 nm and 600 nm were obtained in a Multiskan EX microplate reader. For experiments with multiple time points, plates were kept in a 37 °C incubator between absorbance readings. Controls included media alone, media plus AlamarBlue, media plus AlamarBlue plus berberine dilution, and cells plus media plus AlamarBlue. Percent reduction of AlamarBlue was calculated using the manufacturer’s formula [58], with replacement of their negative control, which contains only media plus AlamarBlue, with a more robust negative control, media plus AlamarBlue plus a drug concentration equal to each experimental well:

$$\frac{\left(\varepsilon \text{0X}\right)\lambda_{2}A\lambda_{1}-\left(\varepsilon \text{0X}\right)\lambda_{1}A\lambda_{2}}{\left(\varepsilon \text{0X}\right)\lambda_{2}A^{\text{o}}\lambda_{1}-\left(\varepsilon \text{0X}\right)\lambda_{1}A^{\text{o}}\lambda_{2}}\times 100$$

In the formula, ελ_1_ and ελ_2_ are constants representing the molar extinction coefficient of AB at 570 and 600 nm, respectively, in the oxidized (ε_ox_) form. *A*λ_1_ and *A*λ_2_ represent absorbance of test wells at 570 and 600 nm, respectively. *A*^0^λ_1_ and *A*^0^λ_2_ represent absorbance of positive control wells at 570 and 600 nm, respectively. The values of %AB reduction were corrected for background values of negative controls containing medium without cells. Assays were performed at least twice, and the average % reduction was used to determine the MIC. AlamarBlue MIC (MIC_AB_) defined as the lowest BECl concentration resulting in ≤50% reduction of AB (average of two experiments) and a purple/blue well 60 min after addition of AB.

### 3.4. Antibacterial Susceptibility Testing; Disk Diffusion Method

All isolates were tested for antimicrobial susceptibility by the disk diffusion method-based analysis, using MHA + 5% sheep blood and commercially available disks containing an antimicrobial agent according to the EUCAST recommendations [56]. For disk diffusion testing, 90 mm plates with the agar medium were inoculated by swabbing the agar with a swab soaked in a bacterial suspension of 1 × 10^8^ cells/mL. Disks (EMAPOL, Gdansk, Poland) containing penicillin (PEN) 1 IU, erythromycin (ERY) 15 µg, clindamycin (DA) 2 µg, oxacillin (OXA) 1 µg, ciprofloxacin (CIP) 5 µg, linezolid (LIN) 10 µg, tetracycline (TET) 30 µg or trimethoprim + sulfamethoxazole (SXT) 1.25 + 23.75 µg were used for the analysis of antimicrobial susceptibility.

The combined effect of antibiotics and BECl was studied using plates with MHA plus 1/4 of MIC_90_ of BECl, which was considered as a sub-inhibitory concentration [59,60]. Disks were placed onto the agar surface and gently pressed to ensure contact using the sterile forceps. Plates were incubated at 35 °C for 20 h in air. The susceptibility testing of each antibiotic for each isolate and the reference strains was performed in triplicates. After the incubation period diameters of the growth inhibition zones (in mm) were measured for each strain, and the mean values were calculated. Those antibiotics have been reported as anti-staphylococcal and anti-streptococcal agents with different target points.

### 3.5. Statistical Analysis

To determine the percentage of the variation attributable to the factors such as bacterial strains; time; and concentrations the results concerning the bacterial growth were analyzed by a three-way analysis of variance (ANOVA). The results from synergism assay were submitted to the Wilcoxon Signed-Rank Test comparing the values (mm) of the inhibitory zone in the disk diffusion method. All statistical analyses were performed using the Statistica 10.0 v software package (Statsoft, Tulsa, OK, USA); assuming the statistical significance level of *p* < 0.05.

## 4. Conclusions

The growth of oral streptococci can be efficiently inhibited by berberine, a biologically active natural substance from medicinal plants, acting alone as a single antibacterial agent. The importance of this study is due to the fact that antibacterial effect of berberine can be enhanced by the synergistic action of common antibiotics. Reduction in oral microflora as an effect of antimicrobial efficacy of berberine-containing measures may deliver an alternative treatment approach for immunocompromised individuals with high risk of opportunistic infection or dental caries and would be a promising agent for oral microbiota control.
